# The role of triglyceride-glucose index in the differential diagnosis of atherosclerotic stroke and cardiogenic stroke

**DOI:** 10.1186/s12872-024-03857-4

**Published:** 2024-06-08

**Authors:** Mengqian Liu, Xiaoyun Yang, Yi Jiang, Wen Zhong, Yiwen Xu, Guanghui Zhang, Qi Fang, Xiaozhu Shen

**Affiliations:** 1 Department of Geriatrics, Lianyungang Hospital Affifiliated to Jiangsu University (Lianyungang Second People’s Hospital), Lianyungang, China; 2 Department of Geriatrics, Bengbu Medical College Clinical College of Lianyungang Second People’s Hospital, Lianyungang, China; 3 Department of Infectious Disease, The First Affiliated Hospital of Ningbo University, Ningbo, China; 4 Department of Neurology, The Affiliated Lianyungang Hospital of Xuzhou Medical University, Lianyungang, China; 5 Department of Neurology, The First Affiliated Hospital of Soochow University, Suzhou, China

**Keywords:** Triglyceride glucose index, Atherosclerotic stroke, Cardiogenic, Insulin resistance stroke

## Abstract

**Objective:**

This study aims to investigate the role of the triglyceride glucose (TyG) index in differentiating cardiogenic stroke (CE) from large atherosclerotic stroke (LAA).

**Method:**

In this retrospective study, patients with acute ischemic stroke were recruited from the First Affiliated Hospital of Soochow University, Lianyungang Second People’s Hospital and Lianyungang First People’s Hospital. Their general data, medical history and laboratory indicators were collected and TyG index was calculated. Groups were classified by the TyG index quartile to compare the differences between groups. Logistic regression was utilized to assess the relationship between the TyG index and LAA. The receiver operating characteristic curve (ROC) curve was used to evaluate the diagnostic efficiency of the TyG index in differentiating LAA from CE.

**Result:**

The study recruited 1149 patients. After adjusting for several identified risk factors, groups TyG-Q2, TyG-Q3, and TyG-Q4 had a higher risk of developing LAA compared to group TyG-Q1(odds ratio (OR) = 1.63,95% confidence interval (CI) = 1.11–2.39, OR = 1.72,95%CI = 1.16–2.55, OR = 2.06,95%CI = 1.36–3.09). TyG has certain diagnostic value in distinguishing LAA from CE(AUC = 0.595, 95%CI0.566–0.623;*P*<0.001).

**Conclusion:**

Summarily, the TyG index has slight significance in the identification of LAA and CE; it is particularly a marker for their preliminary identification.

## Introduction

Stroke is the 3rd leading cause of death globally, and has recently become the major cause of mortality in China with an extremely high disability rate [1, 2]. After stroke, patients experience different degrees of dysfunction, particularly motor dysfunction [3]. Acute ischemic stroke is the most common type of stroke, with the etiological classification of acute ischemic stroke is still based on the TOAST(Trial of Org 10,172 in Acute Stroke Treatment) classification of 1993 [4]. Large artery atherosclerotic (LAA) stroke accounts for 60% of acute ischemic stroke [5]; cardiogenic (CE) stroke accounts for 14–30% and is the most deadly stroke subtype [6], whereas atrial fibrillation related stroke accounts for 79% of cardiogenic strokes [7]. Notably, the CHADS2 score is a scale used to evaluate the risk of stroke in patients with non-valvular atrial fibrillation [8]. Studies have shown that an increase in CHADS2 score increases the risk of embolic events [9]. However, most components of CHADS2 score are also risk factors for atherosclerosis. At the same time, studies have found that approximately 20% of patients with atrial fibrillation have severe carotid artery stenosis [10].

We selected LAA and CE for this research since they cause most types of acute ischemic stroke, despite the presence of partially similar risk factors for both, atrial dysrhythmia and sudden onset to maximum deficit were significant predictors of cardioembolic stroke, whereas hypertension, chronic obstructive pulmonary disease, diabetes, hypercholesterolemia and/or hypertriglyceridemia and age were independent predictors of atherothrombotic stroke in a clinical study aimed to analyze the early differentiation of cardioembolic from atherothrombotic cerebral infarction [11]. Thus, LAA and CE have different secondary prevention strategies. In addition, acute management strategies for different stroke subtypes are also different [12], hence it is important to clearly differentiate between LAA and CE.

Insulin resistance(IR) is a marker of metabolic syndrome and a significant risk factor for stroke [13]. At the same time, it is also a risk factor for recurrent stroke [14], with multiple recurrent strokes resulting in worse outcomes and higher mortality. The triglyceride glucose (TyG)index is a novel marker of insulin resistance, calculated using triglyceride and glucose; it is simple, fast and easy to obtain. Of note, it is more difficult to identify acute ischemic stroke in primary hospitals at an early stage, identify different subtypes, and conduct accurate preliminary treatment. This is attributed to the difficulty in conducting cerebrovascular angiography, computed tomography angiography (CTA), and other examinations in most community hospitals and the lack of experienced neuroimaging doctors. As a result, this may delay the optimal treatment opportunity and significantly affect the prognosis of acute ischemic stroke.

Previous studies indicate that the TyG index is closely associated with the occurrence and prognosis of stroke, and positively correlates with the risk of acute ischemic stroke. Patients with acute ischemic stroke and a higher TyG index have a higher risk of death or a worse prognosis [15]. A 2021 study revealed that patients with atherosclerosis diaplayed an increased TyG index [16], which is associated with the risk of LAA [17].

Additionally, one of our previous studies has found that the TyG index is an independent predictor of carotid plaque formation in people over 40 years of age at high risk of stroke [18]. TyG index is closely related to stroke, particularly LAA. However, 2022 research found that the TyG index is not associated with the incidence of atrial fibrillation [19]. However, studies on the role of the TyG index in differentiating LAA and CE are limited. Thus, this work aims to investigate the role of the TyG index in the differentiation of LAA and CE and offer guiding significance for secondary prevention and treatment of clinical stroke.

## Materials and methods

### Study design and population

The study included acute ischemic stroke patients undergoing treatment at the Stroke Center of the First Affiliated Hospital of Soochow University between March 2018 and January 2021, at the Stroke Center of Lianyungang Second People’s Hospital between January 1, 2022 and July 31, 2023, and at the Stroke Center of Lianyungang First People’s Hospital between October 2019 and December 2021. All three stroke centers are national advanced Stroke Centers. This study was approved by the Ethics Committee of the First Affiliated Hospital of Soochow University, Lianyungang Second People’s Hospital and Lianyungang First People’s Hospital. All the protocols and procedures of this study were implemented according to the Declaration of Helsinki. Informed consent of all subjects was obtained for this study. This study passed the ethical review of the Affiliated Hospital of Soochow University, Lianyungang Second People’s Hospital and Lianyungang First People’s Hospital (Table 1).

**Table 1:** Research institutions and ethics

Institution	Number of cases	Ethics
the First Affiliated Hospital of Soochow University	386	No.2020272,2019057
Lianyungang Second People’s Hospital	354	No.2020050
Lianyungang First People’s Hospital	409	No. KY-20210917001-01

The inclusion criteria included: (1) Age ≥ 18 years old; (2) Meeting the diagnostic criteria for acute ischemic stroke; (3) Voluntary participation of the patient, providing informed consent.

The exclusion included: (1) Intracranial hemorrhage or mass lesion; (2) Severe infection or septic shock; (3) Liver and kidney failure; (4) Incomplete laboratory, clinical data; (5) Small vascular occlusive stroke, unknown cause stroke and unclassified stroke were excluded in TOAST classification.

### Baseline data collection

During enrollment, the subjects were given a history collection and subjected to routine physical examination by an experienced physician. Medical history included age, gender, history of hypertension, diabetes, smoking and drinking. Laboratory tests included creatinine (Cr), homocysteine (Hcy), cholesterol (TC), triglyceride (TG), low density lipoprotein (LDL), high density lipoprotein (HDL), glucose (Glu), and glycosylated hemoglobin (HbA1c).The National Institute of Health stroke scale (NHISS) score was performed by an experienced neurologist blinded to the clinical data.

### Calculation of TyG index

TyG index was calculated as ln[fasting triglyceride (mg/dL) × blood glucose (mg/dL) /2] [20].

### Statistical analysis

Statistical analyses were performed using the R version 4.2.2 (R Core Team 2022). Statistical tests were two-sided. A *P*-value of < 0.05 was considered to be statistically significant. The Kolmogorov–Smirnov test was used to test the normality of the data. For parameters with continuous data, the normal distribution was expressed as mean ± standard deviation, and the skewed distribution was all expressed as median and quartile range (P25–P75). Count data were expressed as a rate (%). Normal distribution data were analyzed by the ANOVA-test, whereas non-normal distribution data were analyzed by the Kruskal–Wallis test. Fisher’s exact test or the Chi-square test was used to compare categorical variables as appropriate. Binary logistic regression analysis was used to assess the role of TyG in the identification of CE and LAA.The receiver operating characteristic curve (ROC) curve was used to evaluate the efficacy of TyG in the identification of CE and LAA. We computed optimal cut-off value and corresponding sensitivity and specificity were calculated.

## Results

**Table 2:** Characteristics of study participants according to TyG index quartiles

Variable	TyG Index quartiles					
	Total (*n* = 1149)	Q1 (*n* = 282)	Q2 (*n* = 291)	Q3 (*n* = 284)	Q4 (*n* = 292)	*P* value
Age, M (Q_1_, Q_3_) (year)	69 (61–76)	73 (65–80)	69 (63–77)	68 (60–74)	66 (56–73)	< 0.001^***^
Gender, n (%)						0.007^**^
female	425 (36.99)	101 (35.82)	86 (29.55)	121 (42.61)	117 (40.07)	
male	724 (63.01)	181 (64.18)	205 (70.45)	163 (57.39)	175 (59.93)	
Smoking, n (%)	334 (29.07)	81 (28.72)	78 (30.58)	78 (27.46)	86 (29.45)	0.870
Drinking, n (%)	270 (21.99)	60 (21.27)	78 (26.80)	58 (20.42)	60 (20.54)	0.197
DM, n (%)	309 (26.89)	30 (10.64)	53 (18.21)	82 (28.87)	144 (49.32)	< 0.001^***^
Hypertension, n (%)	807 (70.23)	165 (58.51)	209 (71.82)	203 (71.48)	230 (78.77)	< 0.001^***^
SBP, M (Q_1_, Q_3_) (mmHg)	150 (138–165)	148 (133–160)	151 (138–168)	151 (137–164)	152 (141–166)	0.003^**^
DBP, M (Q_1_, Q_3_) (mmHg)	86 (78–98)	85 (76–96)	86 (79–99)	86 (79–97)	89 (80–100)	0.011^*^
Hcy, M (Q_1_, Q_3_) (umol/L)	10.5 (8.5–13.2)	11.3 (9.1–14.1)	11.1 (8.8–13.6)	9.8 (8.1–12.6)	10.0 (8.1–12.5)	< 0.001^***^
HbA1c, M (Q_1_, Q_3_) (%)	6.08 (5.60–7.30)	5.80 (5.50–6.00)	5.90 (5.60–6.30)	6.30 (5.80–7.50)	7.80 (6.00–9.23)	< 0.001^***^
Cr, M (Q_1_, Q_3_) (mmol/L)	65 (54–78)	66 (56–80)	68 (57–80)	62 (51–74)	62 (50–76)	< 0.001^***^
Glu, M (Q_1_, Q_3_) (mmol/L)	5.91 (5.08–7.62)	5.01 (4.62–5.55)	5.54 (4.98–6.33)	6.45 (5.64–8.05)	8.68 (6.45–11.49)	< 0.001^***^
TC, M (Q_1_, Q_3_) (mmol/L)	4.44 (3.77–5.15)	3.95 (3.36–4.56)	4.33 (3.81–4.92)	4.68 (3.93–5.36)	4.84 (4.04–5.54)	< 0.001^***^
TG, M (Q_1_, Q_3_) (mmol/L)	1.26 (0.93–1.83)	0.78 (0.65–0.92)	1.16 (0.98–1.30)	1.51 (1.21–1.80)	2.48 (1.84–3.40)	< 0.001^***^
HDL, M (Q_1_, Q_3_) (mmol/L)	1.05 (0.91–1.22)	1.07 (0.93–1.31)	1.06 (0.93–1.22)	1.04 (0.91–1.22)	1.03 (0.88–1.16)	0.003^**^
LDL, M (Q_1_, Q_3_) (mmol/L)	2.67 (2.13–3.19)	2.33 (1.81–2.72)	2.68 (2.16–3.10)	2.91 (2.31–3.42)	2.85 (2.34–3.39)	< 0.001^***^
TOAST, n (%)						< 0.001^***^
CE	253 (22.02)	88 (31.21)	61 (20.96)	56 (19.72)	48 (16.44)	
LAA	896 (77.98)	194 (68.79)	230 (79.04)	228 (80.28)	244 (83.56)	
NIHSS admission, M (Q_1_, Q_3_)	5 (2–10)	5 (2–10)	4 (2–10)	5 (2–10)	5 (2–10)	0.955
NIHSS at discharge, M (Q_1_, Q_3_)	3 (1–8)	2 (1–7)	2 (1–8)	3 (1–9)	3 (1–8)	0.609

SBP, systolic blood pressure; DBP, diastolic blood pressure; Hcy, homocysteine; HbAlc, glycosylated hemoglobin; Cr, creatinine; Glu, glucose; TC, cholesterol; TG, triglyceride; HDL, high density lipoprotein; LDL, low-density lipoprotein; TOAST, Trial of Org 10,172 in Acute Stroke Treatment; CE, cardiogenic; LAA, large artery atherosclerotic; NHISS, National Institute of Health stroke scale; *p < 0.05; **p < 0.01; ***p < 0.001

**Fig. 1:**
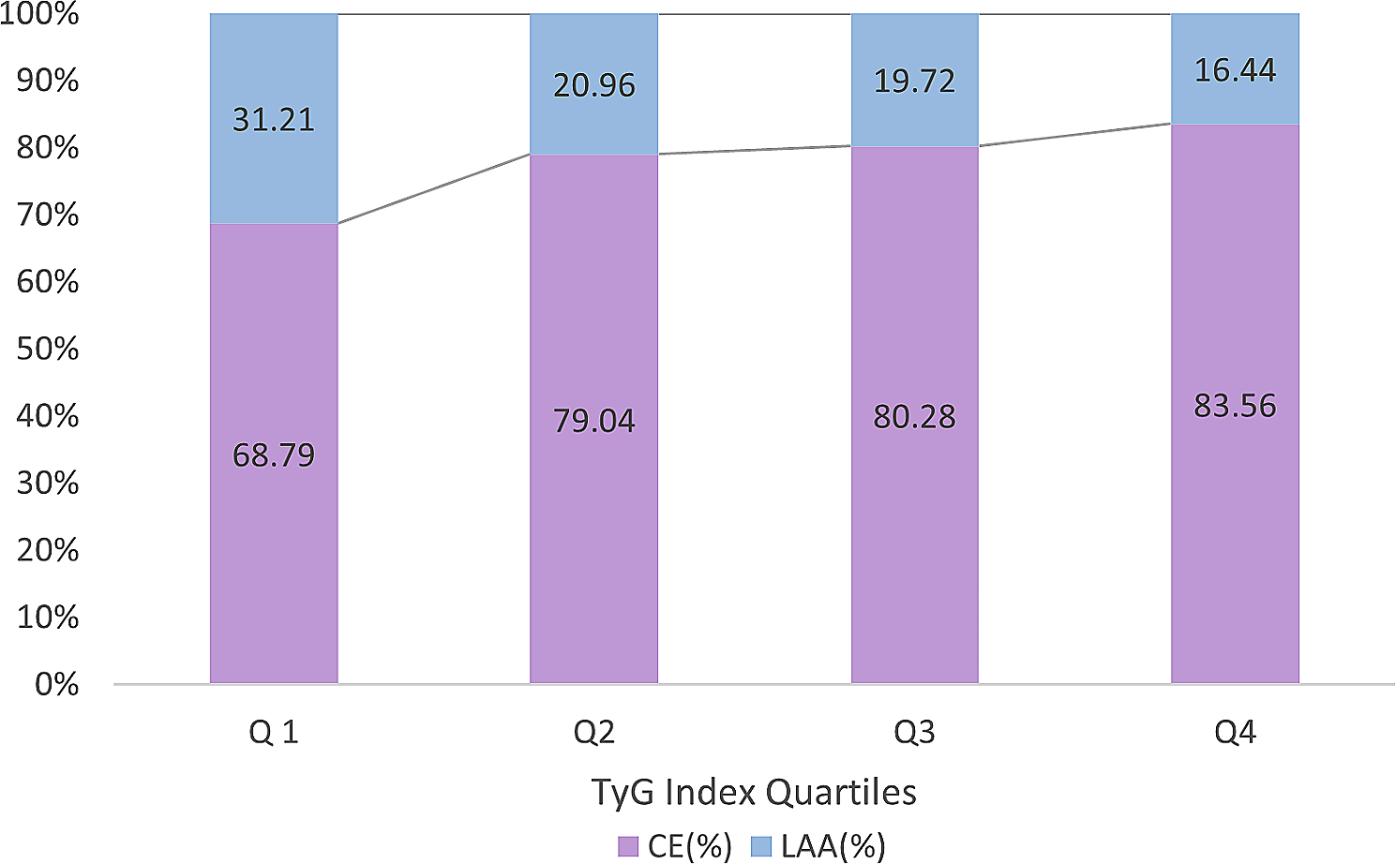
LAA ratio and CE ratio of TyG quartiles The proportion of LAA in TyGQ1, Q2, Q3 and Q4 groups gradually increased, while the proportion of CE gradually decreased; LAA, large artery atherosclerotic; CE, cardiogenic; TyG, triglyceride glucose

**Table 3:** Univariate and multivariate logistic analysis of factors associated with LAA

Model	TyG Index quartiles				Positive predictive value	Negative predictive value	diagnostic accuracy	*P* value
	Q1	Q2	Q3	Q4				
1	Reference	1.71 (1.17–2.50) ^**^	1.85 (1.26–2.72) ^**^	2.31 (1.55–3.44) ^***^	80.99	31.19	68.76	< 0.001 ^***^
2	Reference	1.63 (1.11–2.39) ^*^	1.72 (1.16–2.55) ^**^	2.06 (1.36–3.09) ^***^	85.07	28.77	56.31	< 0.001 ^***^
3	Reference	1.62 (1.10–2.37) ^*^	1.73 (1.16–2.56) ^**^	2.06 (1.37–3.10) ^***^	84.71	30.27	60.31	< 0.001 ^***^
4	Reference	1.48 (1.01–2.19) ^*^	1.53 (1.02–2.29) ^*^	1.66 (1.07–2.56) ^*^	86.56	30.29	57.96	0.023 ^*^
5	Reference	1.51 (1.02–2.24) ^*^	1.58 (1.04–2.41) ^*^	1.96 (1.18–3.25) ^**^	89.07	31.48	58.05	0.009^**^

Model 1: Unadjusted

Model 2: Adjusted age, gender

Model 3: Adjusted for age, gender, smoking history, drinking history

Model 4: Adjusted for age, gender, smoking history, drinking history, hypertension history, diabetes history

Model 5: Adjusted for age, gender, smoking history, drinking history, hypertension history, diabetes history, SBP, DBP, Cr, Glu, HDL, Hcy, HbA1c

^*^P<0.05;^**^P<0.01;^***^P<0.001

**Table 4:** Univariate and multivariate logistic subgroup analysis of LAA related factors

Group	TyG quartiles				*P* value
	Q1	Q2	Q3	Q4	
Male	Reference	1.82 (1.11–2.97) ^*^	1.86 (1.07–3.23) ^*^	2.51 (1.33–4.75) ^**^	0.005^**^
Famale	Reference	1.25 (0.63–2.47)	1.76 (0.91–3.41)	2.17 (0.95–4.93)	0.065
DM	Reference	4.43 (1.40–13.99) ^*^	4.24 (1.42–12.64) ^*^	5.90 (1.83–19.05) ^**^	0.003 ^**^
Non-DM	Reference	1.41 (0.92–2.16)	1.57 (0.99–2.50)	1.97 (1.12–3.46) ^*^	0.019 ^*^
Elderly	Reference	1.56 (1.02–2.38)^*^	1.77 (1.11–2.82) ^*^	2.33 (1.31–4.13) ^**^	0.004 ^**^
Non-elderly	Reference	2.73 (0.82–9.08)	2.32 (0.78–6.91)	3.45 (0.94–12.67)	0.062

Adjusted for age, gender, smoking history, drinking history, hypertension history, diabetes history, SBP, DBP, Cr, Glu, HDL, Hcy, HbA1c; ^*^P<0.05; ^**^P<0.01

### Characteristics of study participants according to TyG index quartiles

A total of 1149 patients were enrolled in this study and divided into four groups following the TyG Index quartile. Table 2 summarizes the clinical features of all study subjects. Unlike CE, LAA had a higher proportion of patients in the Q1, Q2, Q3, and Q4 groups (*P* < 0.001) (Table 2) and the proportion of LAA in Q1, Q2, Q3 and Q4 groups gradually increased (Fig. 1). Among the four groups,

significant differences were noted in age, sex, history of hypertension, history of diabetes, as well as levels of SBP, DBP, Hcy, HbA1c, Cr, Glu, TG, TC, HDL and LDL.

### Univariate and Multivariate Logistic Analysis of Factors Associated with LAA

The relationship between the TyG Index level and its occurrence was analyzed with LAA acting as the dependent variable. Table 3 shows the results. Binary logistic regression analysis model 1 revealed that participants in the TyG-Q2, TyG-Q3, and TyG-Q4 groups had a higher risk of developing LAA unlike those in the TyG-Q1 group (Model 2: odds ratio (OR) = 1.63,95% confidence interval.

(CI) = 1.11–2.39, OR = 1.72,95%CI = 1.16–2.55, OR = 2.06,95%CI = 1.36–3.09;positive predictive value = 85.07,negative predictive value = 28.77,diagnostic accuracy = 56.31, *P*<0.001). After adjusting for age, sex, smoking, alcohol consumption, hypertension, diabetes, SBP, DBP, Cr, Glu, HDL, Hcy and HbA1c, compared with TYG-Q1, the high risk of LAA for TyG-Q2, TyG-Q3, and TyG-Q4 remains.

### Univariate and multivariate logistic subgroup analysis of LAA related factors

Subsequently, we performed a subgroup analysis on gender, diabetes history and age. For male patients, LAA was more likely to occur in Q2, Q3, and Q4 groups than in Q1(OR = 1.82,95%CI = 1.11–2.97; OR = 1.86, 95% CI = 1.07–3.23; OR = 2.51,95%CI = 1.33–4.75; *P* = 0.005) (Table 4). For patients with diabetes, LAA was more common in Q2, Q3 and Q4 groups than in Q1 group (OR = 4.43,95%CI = 1.40-13.99; OR = 4.24, 95% CI = 1.42–12.64; OR = 5.90,95%CI = 1.83–19.05; *P* = 0.003) (Table 4). For elderly patients, LAA was more common in Q2, Q3 and Q4 groups than in Q1 group (OR = 1.56,95%CI = 1.02–2.38; OR = 1.77, 95% CI = 1.11–2.82; OR = 2.33,95%CI = 1.31–4.13; *P* = 0.004) (Table 4).

### ROC analysis testing the capacity of the TyG index to distinguish LAA from CE

**Fig. 2:**
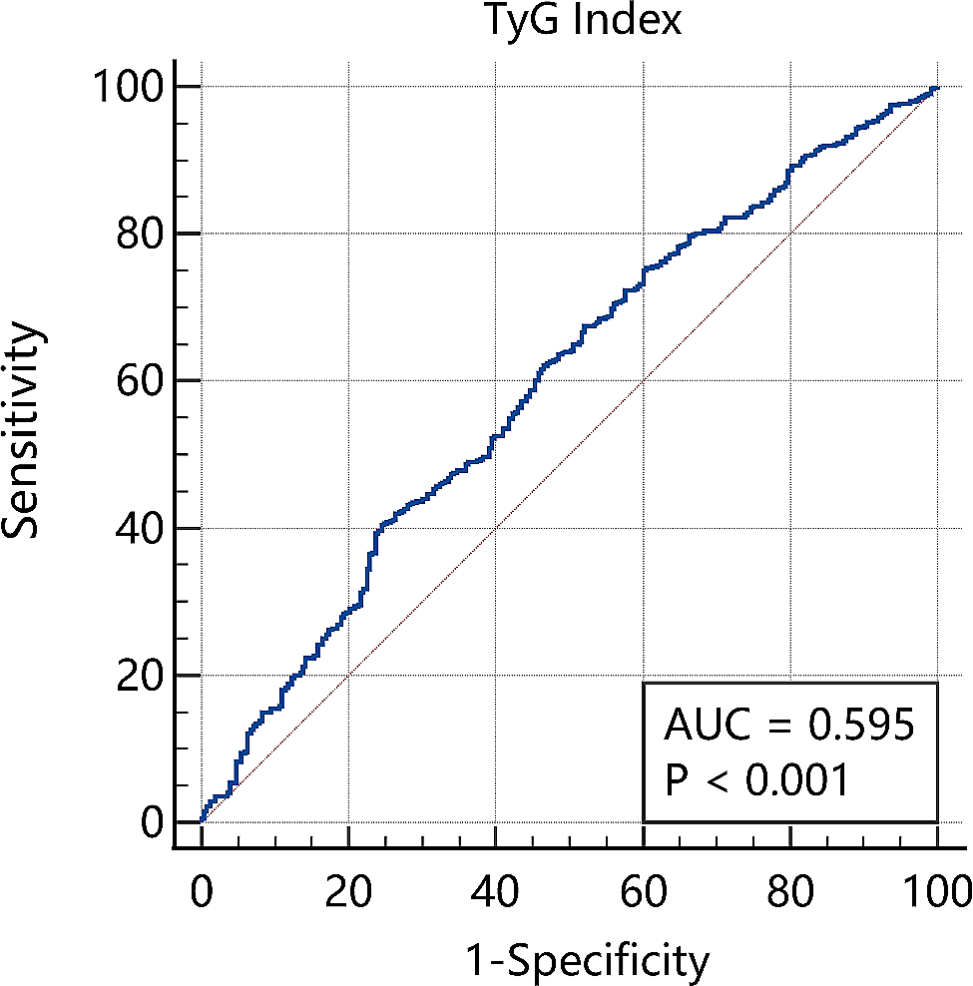
ROC analysis evaluates the ability of the TyG index to distinguish LAA and CE, with an AUC of 0.595 (95% CI 0.566 to 0.623), respectively. The optimal cutoff value of the TyG index was 8.98. Youden index = 0.160, sensitivity = 75.49%, specificity = 40.51%

ROC curve results showed that TyG had a certain diagnostic value in the differentiation of CE and LAA, with the area under the curve (AUC) of 0.595 (95%CI 0.5666–0.623; *P* < 0.001)(Fig. 2). The ROC analysis also showed 8.98 as the optimal cut of the value of the TyG index in distinguishing LAA and CE, with a Youden index of 0.160, sensitivity of 75.49%, and specificity of 40.51%.

## Discussion

This is arguably the first study to assess the role of the TyG index in differentiating large atherosclerotic stroke from cardiogenic stroke. We noted that the higher the TyG index, the higher the proportion of patients suffering from LAA. The regression analysis model showed that the TyG index is related to the risk of LAA occurrence; this relationship still existed after adjusting for related factors. Additionally, ROC curve analysis showed that TyG helped distinguish between LAA and CE subtypes.

The clinical identification of LAA and CE is primarily based on comprehensive clinical judgment including medical history, symptoms and signs, and imaging examination, and requires experienced physicians. However, these are often not enough in actual clinical work. Current guidelines, recommend antiplatelet aggregation drugs for LAA, and anticoagulants for CE [21]. Early etiology identification is of great significance for secondary prevention, treatment and recurrence of stroke patients. Therefore, simple, as well as easily available laboratory indicators are helpful in the identification of LAA and CE.

Glucose metabolism disorder plays an important factor in the occurrence and development of atherosclerosis. Increased blood sugar levels cause endothelial dysfunction, which in turn causes white blood cell infiltration and accumulation of blood vessel walls [22], resulting in the formation of atherosclerotic plaques. In addition, persistent high blood sugar may cause the development of atherosclerosis. A 2017 experimental animal study revealed that the blood vessels of rabbits with diabetes did not increase in hypoxia-inducing factor 1(HIF-1) in the absence of oxygen, whereas HIF-1 can reduce cell death by reducing oxidative stress, mitochondrial damage, and metabolic disorders [23]. This suggests that the blood vessels of diabetic rabbits are less responsive to hypoxia, thus prolonged hypoxia causes cell death, increased necrotic cores, and accelerated atherosclerosis progression [24]. Abnormal blood sugar often aggravates dyslipidemia, which is often secondary to insulin resistance or related factors [25]. In addition, triglyceride accumulation can influence the arteries via insulin resistance and promote the production of inflammatory factors to harden them [26]. Therefore, hyperglycemia and hyperlipidemia interact and jointly promote the occurrence and development of atherosclerosis.

Insulin resistance contributes to the occurrence of atherosclerosis through metabolic abnormalities [27], which can cause endothelial dysfunction and increase the advanced glycosylation end products, thereby resulting in atherosclerosis [28, 29]. Homeostasis model assessment of insulin resistance (HOMA - IR) is one of the most commonly used methods to assess insulin resistance index in clinical practice [30]. A study in 2008 first proposed that the TyG index is a reliable marker of insulin resistance [31]. Recent studies indicate that the TyG index is superior to HOMA in the assessment of atherosclerosis [32, 33]. Therefore, several studies have focused on the relationship between the TyG index and atherosclerosis. Cerebral hypoperfusion caused by atherosclerosis and plaque rupture embolism are the main causes of LAA [34, 35]. A study in 2021 [16] reported that the TyG index is an independent risk factor for atherosclerosis in both linear regression and logistic regression. Moreover, in a different study in the same year, the TyG index accelerates the instability of atherosclerotic plaques by targeting innate immune active and inflammatory active substances, thereby causing LAA [36]. Moreover, subsequent studies confirmed the relationship between the TyG index and LAA. In line with our findings, Jiang et al. discovered that the TyG index positively correlates with a high incidence of LAA [17].

According to the present study, the TyG index of male, diabetic patients and elderly patients played a more significant role in the identification of CE and LAA than that of female, non-diabetic patients and non-elderly patients; this may be attributed to the lower insulin sensitivity of male, diabetic patients and elderly patients. Although one of the inclusion criteria was ≥ 18 years of age, Table 1 shows that most of the study participants aged approximately 60 years, and gonadal function had begun to decrease in most men. Studies indicate that testosterone plays an important role in regulating insulin sensitivity, and low levels of testosterone can increase insulin resistance [37]. Moreover, studies have found that smoking is an independent risk factor for insulin resistance [38], and 97.6% of the smoking population in our study was male, which may also explain the gender difference. Similarly, most of the diabetic patients included in our study were type 2 diabetics, with insulin resistance [39].

This study is a multicenter study, which increases the generalizability of findings. The results show that the TyG index may be a promising marker for the identification of LAA and CE, particularly playing a key role in early identification. Besides, the TyG index can help in the secondary prevention of stroke, the selection of appropriate treatment plans, and in controlling poor prognosis, recurrence rate, and mortality of stroke. At the same time, the TyG index is simple and easy to obtain, which is extremely important in the differentiation of CE and LAA in primary hospitals.

The present study has limitations. First, this was a cross-sectional study, and could not investigate the potential mechanism by which the TyG index differentiates LAA from CE. Secondly, we only focused on the two factors of gender and diabetes history in the subgroup analysis, yet other factors may also influence the role of the TyG index in the identification of LAA and CE. Thus, this has to be confirmed by subsequent studies.

Our study found that TyG may contribute to the differentiation of stroke LAA and CE subtypes, but its sensitivity and specificity are not very satisfactory. Therefore, we will study the role of TyG-related indicators including TyG-BMI (body mass index), TYG-WC(waist circumference), TYG-WHTR(waist-to height ratio) and other TYG-related indicators in the differentiation of LAA and CE in the future.

## Conclusion

In conclusion, the TyG index is slightly significant in the identification of LAA and CE; it is specifically a marker for their preliminary identification.

## Data Availability

The datasets used and/or analyzed during the current study are available from the corresponding author on reasonable request.
